# Predator traits influence uptake and trophic transfer of nanoplastics in aquatic systems–a mechanistic study

**DOI:** 10.1186/s43591-024-00096-4

**Published:** 2024-10-09

**Authors:** Amy Ockenden, Denise M. Mitrano, Melanie Kah, Louis A. Tremblay, Kevin S. Simon

**Affiliations:** 1 School of Environment, The University of Auckland, Science Centre, Building 302, 23 Symonds Street, Auckland CBD, Auckland, 1010 New Zealand; 2 ETH Zurich, Department of Environmental Systems Science, Universitatstrasse 16, Zurich, 8092 Switzerland; 3 School of Biological Sciences, The University of Auckland, Building 110, 3A Symonds Street, Auckland CBD, Auckland, 1010 New Zealand; 4 Manaaki Whenua-Landcare Research, Lincoln, 7640 New Zealand

**Keywords:** Macroinvertebrate, Contaminant, Feeding, Freshwater, Ecosystem, Uptake, Elimination, Exposure

## Abstract

Predicting the response of aquatic species to environmental contaminants is challenging, in part because of the diverse biological traits within communities that influence their uptake and transfer of contaminants. Nanoplastics are a contaminant of growing concern, and previous research has documented their uptake and transfer in aquatic food webs. Employing an established method of nanoplastic tracking using metal-doped plastics, we studied the influence of biological traits on the uptake of nanoplastic from water and diet in freshwater predators through two exposure assays. We focused on backswimmers (*Anisops wakefieldi*) and damselfly larvae (*Xanthocnemis zealandica*) - two freshwater macroinvertebrates with contrasting physiological and morphological traits related to feeding and respiration strategies. Our findings reveal striking differences in nanoplastic transfer dynamics: damselfly larvae accumulated nanoplastics from water and diet and then efficiently eliminated 92% of nanoplastic after five days of depuration. In contrast, backswimmers did not accumulate nanoplastic from either source. Differences in nanoplastic transfer dynamics may be explained by the contrasting physiological and morphological traits of these organisms. Overall, our results highlight the importance and potential of considering biological traits in predicting transfer of nanoplastics through aquatic food webs.

**Supplementary Information:**

The online version contains supplementary material available at 10.1186/s43591-024-00096-4.

## Introduction

Microplastics (MP) are ubiquitous contaminants with well-established risks to organisms and associated ecosystem processes [1, 2]. A more recent ecological concern stems from fragmentation of environmental plastics into nanoplastics (NP), which are considered an extension of the MP issue [3]. However, because of their small size (< 1000 nm, though some studies define NPs as being < 100 nm [4, 5]) and higher surface area to volume ratio, NP have different modes of toxicity compared to MP, including the potential to permeate biological membranes and accumulate within internal tissues [6].

To assess ecological risks of NP, we must understand their dynamics in aquatic food webs and factors driving their uptake and trophic transfer. NP can enter aquatic food webs by direct uptake from the surrounding environment [7, 8] and indirectly through predator-prey/feeding interactions [9–12]. However, the relative contributions of these pathways to NP uptake in organisms is unknown. Furthermore, there is limited understanding of the biological/physiological traits that render organisms susceptible to NP uptake.

Aquatic ecosystems contain a diverse community of species, each with unique biological and physiological traits. This diversity presents a major challenge in assessing the risks of environmental contaminants, as these traits can influence the uptake and accumulation and, subsequently, the response of organisms [13–15]. For instance, certain organisms with specialized respiratory structures such as gills, spiracles, thin cuticles, and high membrane permeability have an enhanced capacity for *direct* uptake of dissolved contaminants [15, 16]. A similar pattern is observed with particulate contaminants such as NPs, where species with large gills, such as the freshwater bivalve *Corbicula fluminea*, have been shown to bioaccumulate NPs [17, 18]. Traits can also influence the *indirect* transfer of contaminants through trophic interactions, driven by feeding strategy [13]. For example, filter-feeding *Daphnia*, are particularly vulnerable because they cannot discriminate between phytoplankton and non-food particles, such as plastics [19, 20]. Likewise, the feeding strategy of a predator may influence their uptake of contaminants from prey [13, 21]. While previous field and lab studies have highlighted the influence of certain traits, e.g., feeding strategy, on MP uptake from water [22, 23], equivalent studies on NP are lacking.

**Fig. 1:**
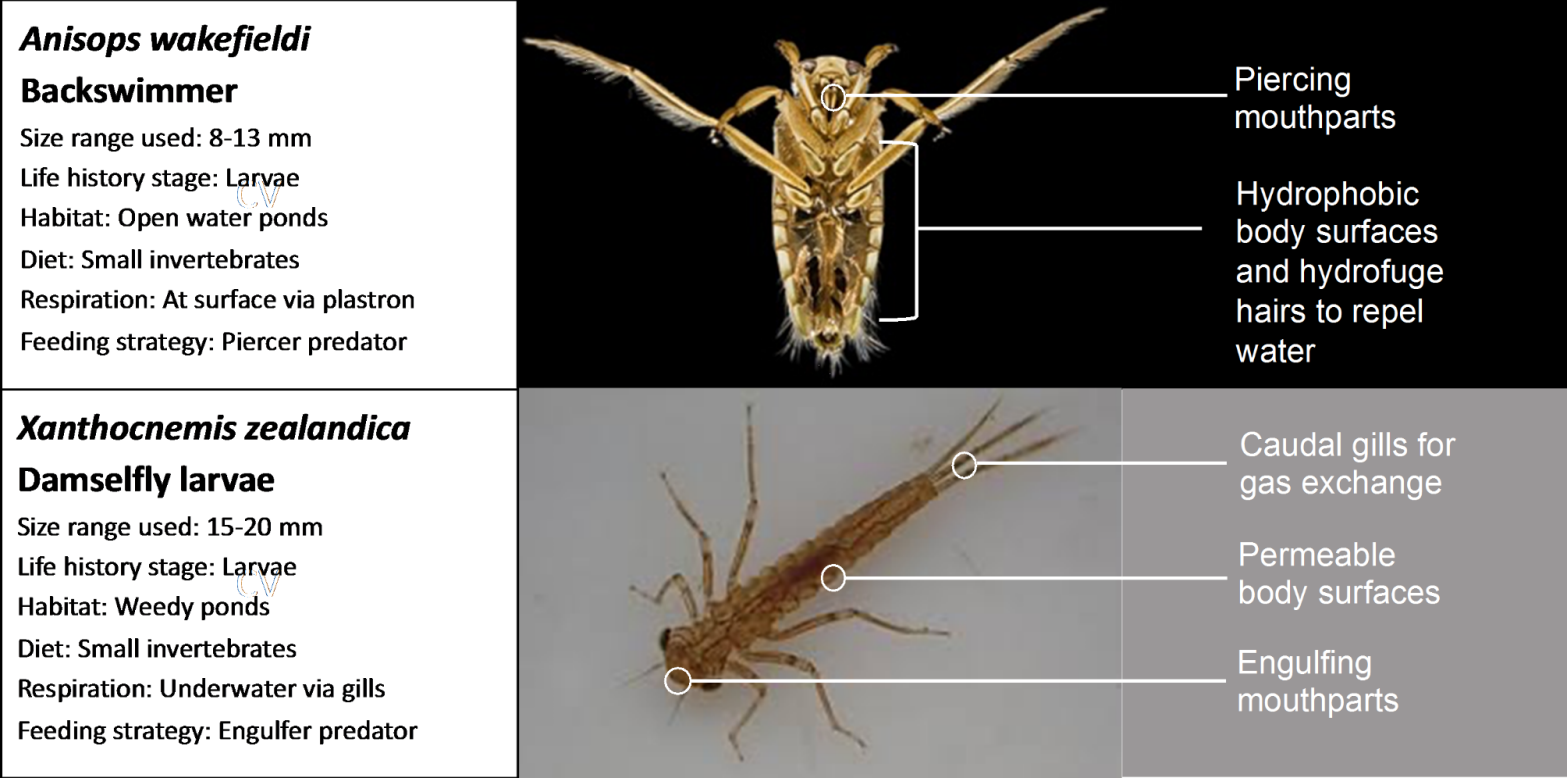
Visual representation of the two predator test organisms, backswimmers (*Anisops wakefieldi*) and damselfly larvae (*Xanthocnemis zealandica*). Details on their contrasting physiological and ecological characteristics which may influence contaminant uptake are included. Images were obtained from Wikimedia commons

In this study, we quantified the relative importance of different NP exposure routes for aquatic organisms and evaluated the influence of physiological traits on NP uptake rates. We first assessed the relative importance of direct (water) and indirect (diet) exposure routes for NP uptake by two predators. Second, we evaluated the influence of predator traits on the trophic transfer and accumulation of NP from diet. To achieve this, we selected two aquatic invertebrate predators with contrasting traits (Fig. 1): backswimmers (*Anisops wakefieldi*), which are piercer-predators and respire using spiracles at the water surface and red damselfly larvae (*Xanthocnemis zealandica*), which are engulfer-predators and respire using gills. To examine trophic transfer, we selected *Daphnia magna* as a prey species because they accumulate high body burdens of NP [24] and are consumed by a wide range of predators. To accurately quantify NP body burdens over time, we used polystyrene NP doped with a palladium (Pd) tracer [25]. Our overall objective was to investigate the influence of species traits on NP uptake and depuration, enabling us to generate more informed, environmentally relevant hypotheses on the relative susceptibility of different organisms to a major emerging pollutant.

## Results and discussion

### Rapid NP uptake in prey and contrasting uptake rates between predators from the water column

**Fig. 2:**
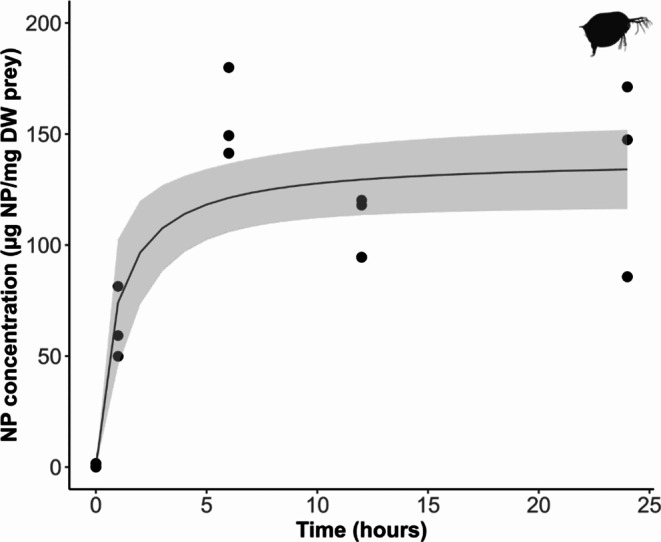
Uptake of NP (µg NP/mg DW) by *Daphnia* over 24-h from water (Exposure 1). Data points are individual replicates (20 individual *Daphnia* per replicate) at each time point (0, 1, 6, 12, 24 h). The solid curve represents a Michaelis-Menten model fitted to the data, and shading represents the lower and upper 95% confidence intervals

*Daphnia* rapidly accumulated NP from the water until reaching a plateau (Fig. 2) and becoming fully saturated with NP after ~ 6 h. This aligns with previous research, which shows *Daphnia* attain full saturation in 4–8 h when exposed to 100–200 nm NP [26, 27]. The maximum saturation concentration (*C*_*max*_) was 138.96 ± 9.75 µg NP/mg DW and *Daphnia* accumulated approximately 20% of the total NP present in the test system over 24-h. The rapid accumulation and high body burden of NP in *Daphnia* can be attributed to their non-selective filter feeding behaviour, as they have limited ability to reject unwanted particles [19, 28]. While it is possible that NP might penetrate or adhere to the external surfaces of *Daphnia* in addition to being consumed [29, 30], our study was not designed to disentangle these routes of exposure.

**Fig. 3:**
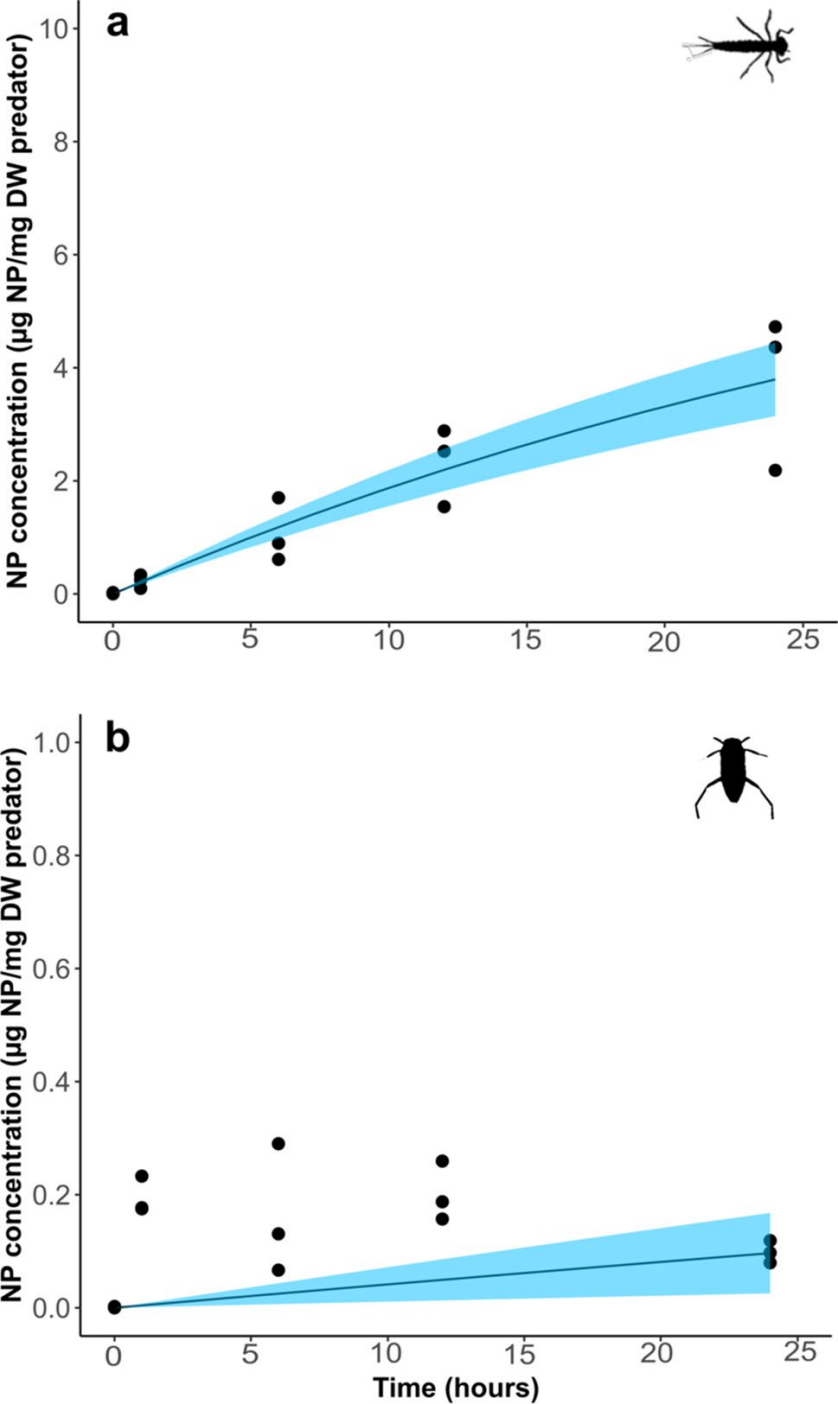
Uptake of NP (µg NP/mg DW) over time in damselfly larvae (**a**) and backswimmers (**b**) when exposed to NP in water (Exposure 1). Data points represent individual replicates (*n* = 1 invertebrate per replicate) at each time point. Solid lines are bioaccumulation models fitted to the data using Eq. 1 and the shading represents upper and lower 95% confidence intervals. Note the y-axes are one order of magnitude different between (**a**) and (**b**)

Damselflies had an uptake rate constant (*k*_*w*_) 500 times higher than that for backswimmers (Fig. 3; Table 1). Damselfly larvae accumulated NP consistently over time (Fig. 3a), reaching 3.76 ± 1.37 µg NP/mg DW after 24-h, representing approximately 0.35% of total available NP. In contrast, backswimmers accumulated negligible concentrations of NP (< 0.3 µg NP/mg DW) and showed no consistent increase over time (Fig. 3b). This pattern suggests that the NPs are likely not being taken up internally. Instead, the NPs may be adhering to external surfaces, such as becoming trapped among external surface features like hydrophobic “hairs,” rather than being absorbed into the organism’s tissues. Difference in NP uptake between predators may result from differences in physiological traits, particularly the mode of respiration and associated morphological features. Damselfly larvae extract oxygen from water through large, highly vascularized gills on their abdominal segments which are water permeable [31], potentially facilitating passive NP uptake through the gills. Conversely, backswimmers respire through spiracles (body openings) covered by a plastron (air bubble), isolating the spiracles from water contaminants [21]. Furthermore, many backswimmer body surfaces are covered in tiny hydrophobic hairs [32, 33], making them water impermeable [34]. While NP distribution in the water column was not measured in this study, previous research using the same NPs at similar concentrations (6 mg/L) in freshwater microcosms found that ~ 90% of the NPs remained suspended in the water column after 48 h [35], a duration longer than our exposure period. Additionally, although backswimmers spend some time at the water surface, they are known to move throughout the water column, which would have brought them in contact with the NPs [36]. Thus, the observed low uptake/accumulation of NPs in backswimmers is likely attributed to their physiological traits. Our findings align with previous studies that show differential uptake of *dissolved* contaminants in organisms with diverse traits. This suggests that while the specific nature of the organism-contaminant interaction may differ depending on whether the contaminant is dissolved or particulate [37], the overarching trend remains consistent. For example, gill-breathing amphipods (*Gammarus pulex*) exhibited uptake rates of pharmaceuticals 8–27 times higher compared to the air-breathing backswimmer *Notonecta glauca* [21]. Similarly, surface-breathing species such as *Notonecta kirvyi* and *Ptychoptera* sp. had the lowest uptake of the pesticide chlorpyrifos among ten tested invertebrates [34].

**Table 1:**
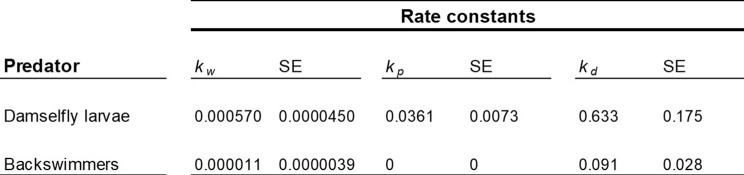
Uptake rate constants from water (*k*_*w*_, L/mg/d) and prey (*k*_*p*_, mg prey/mg predator/d) and elimination rate constants (*k*_*d*_, d^− 1^) and their standard errors (SE) for damselfly larvae and backswimmers

### Predators differ in their uptake of NP from prey

Damselfly larvae consumed an average of 7.5 *Daphnia* per day (out of a maximum of 20 available *Daphnia*), leading to a gradual accumulation of NP in their bodies (Fig. 3a) and an uptake rate constant from prey (*kp*) of 0.0361 (Table 1). In contrast, despite consuming about twice as many prey (average of 16.6 out of 20 *Daphnia* per day), backswimmers did not accumulate any detectable amount of NP from their prey (*kp* = 0) (Fig. 3b; Table 1). For damselflies, water and prey contributed nearly equally to NP uptake, accounting for 52% and 48%, respectively.

Differences in NP accumulation from prey may be attributed to the distinct feeding strategies of these predators [13]. Damselflies consume their prey whole, ingesting internal and external parts. Backswimmers pierce their prey and suck out only the internal fluids [38]. NP mainly accumulate in the digestive tract and external body surface of *Daphnia*, with limited transfer to other body tissues [27, 29, 39, 40] so backswimmers likely did not ingest the tissues of *Daphnia* that accumulated NP. Trophic transfer therefore depends on where NP accumulate in prey and what tissues predators consume.

### Rapid depuration of NP by damselfly larvae

**Fig. 4:**
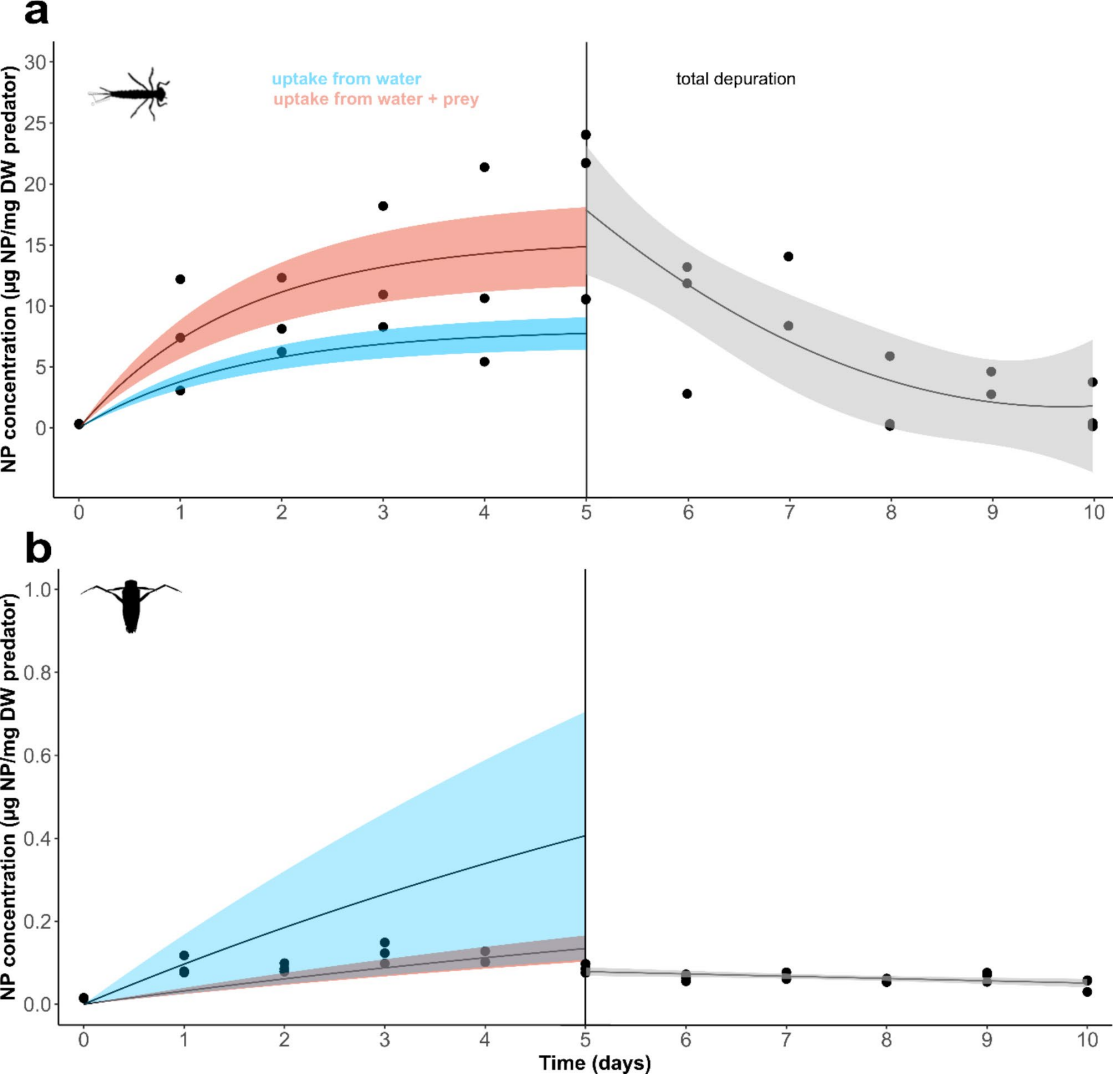
Uptake and depuration of NP (µg NP/mg DW) by damselfly larvae (**a**) and backswimmers (**b**). The left-hand panel depicts the 5-day exposure phase with the modelled total uptake of NP from water and prey combined in red using data generated from Exposure 2. The blue curve is the estimated contribution of direct uptake from water alone based on 24 h Exposure 1 trials. The grey area in (**b**) indicates where the red and blue areas overlap. The right-hand panel shows the 5-day depuration phase. The data points are individual replicates (*n* = 1 invertebrate per replicate) at different time points. Uptake and depuration phases were modelled separately: solid lines represent bioaccumulation models fitted to the data using Eq. 3 for the uptake phase and Eq. 1 for the depuration phase. Shading represents upper and lower 95% confidence intervals

Damselfly larvae rapidly eliminated NP, achieving 92% depuration after 5 days (*k*_*d*_=0.633 d^− 1^) (Table 1; Fig. 4a). Other aquatic organisms have rapid depuration rates of NP, including marine scallops, which eliminated 68% within 3 days when exposed to NP of similar size to ours (250 nm) [41], and oysters which eliminated 92% of 164 nm NP from the digestive gland over 30 days [42]. Likewise, rainbow trout eliminated all NP from their tissues after a 7-day depuration period when exposed to 205 nm NP [43].

Compared with damselflies, backswimmers exhibited substantially slower elimination of NP (*k*_*d*_=0.091) (Fig. 4b). This contrasts with previous studies on *dissolved* contaminants, where backswimmers rapidly eliminated benzophenone and pharmaceutical compounds [13, 21]. It is possible that backswimmers did not actively uptake any NP internally. Instead, NP may have simply adhered to external body parts making physiological excretion impossible. Indeed, the initial concentration of NPs at Day 0 (0.015 µg NP/mg DW) was not substantially different from the concentration at the end of the experiment (Day 10) (0.048 µg NP/mg DW) suggesting minimal internal uptake. While no major outliers were observed in our study, we recorded slight variations in the uptake and depuration of NPs among individual organisms. These differences reflect the natural variation inherent in communities of field organisms and are likely attributable to slight differences in hunger levels, size, and other physiological processes, such as ingestion/egestion rate.

## Conclusions

The influence of biological traits on NP dynamics in freshwater food webs have been largely overlooked. Uptake and effects of NP have been investigated across a broad range of taxonomic groups [1, 2, 44], but it is unclear which traits of organisms influence their NP uptake, and whether these effects are conserved across different taxonomic groups. Our study provides evidence that physiological and morphological traits, such as feeding mode, respiration strategy, and external surface features (e.g., gills, hydrophobic body surfaces), may be more reliable predictors of NP uptake and trophic transfer than an organism’s trophic level alone. Testing every organism for NP uptake is impractical; thus, identifying and understanding the impact of these traits can improve our ability to predict NP behaviour across food webs and guide the development of more accurate ecological models. This approach also enables us to better understand which ecological processes, such as predator-prey interactions, will be most influential in shaping NP dynamics. Direct ingestion of NPs, particularly in species with permeable surface features (e.g., gills) or specialized feeding adaptations like filter-feeders, may be a more significant route of NP uptake. However, indirect uptake through prey can be important for some animals, highlighting the need to consider both routes when modelling NP dynamics. This distinction is important because particulate contaminants like NPs typically enter organisms through ingestion, unlike soluble contaminants that diffuse more passively. We acknowledge that there are a multitude of factors that may influence NP uptake and depuration in the natural environment. Factors such as particle characteristics (size, shape, polymer type, density) can influence NP dynamics; for example, organisms of different sizes exhibit preferences for specific NP sizes [22]. Additionally, variations in exposure conditions, including pH, temperature and natural organic matter concentration, can alter the fate of particles, influencing aggregation and settling rates of NP [45, 46]. Nevertheless, our results suggest that examining animal traits should increase understanding of NP dynamics and improve models designed to predict NP transfer through food webs.

## Methods

### Nanoplastics

A suspension of metal-doped polystyrene (PS) NPs were synthesized according to previously published methods [25]. These NPs consisted of a PS outer shell and a polyacrylnitrile (PAN) core with chemically entrapped palladium (Pd). This NP structure ensured no PAN or Pd was present on the particle surface [25]. Polystyrene is one of the highest-produced plastic polymers [47] and one of the most common polymers identified in NP samples from environmental freshwaters [48, 49]. NPs had a hydrodynamic diameter of 256.4 ± 1.5 nm (polydispersity index = 0.113) and a zeta potential of -32.1 ± 4.57 mV determined using dynamic light scattering with a Malvern Zetasizer in ultrapure water (Figure S1). Total Pd concentration in the suspension was 73.1 mg/L, confirmed using inductively coupled plasma mass spectrometry (ICP-MS), and particle concentration was determined to be 25,975 mg/L, measured by drying 2 mL of suspension at 60 °C for 48 h. Thus, the Pd mass fraction of the NP was approximately 0.28% (w/w) (Supplementary text 1). The density of the model NPs is not significantly affected by Pd inclusion, and the low Pd content is not expected to affect the study results. Plastics and NPs often contain metal additives (e.g., heat stabilizers, colorants, antioxidants), implying that environmental NPs can have varying densities even with the same base polymer.

### Study organism collection and maintenance

Adult *Daphnia magna* from a commercial aquarium supplier were housed in 15 L aquaria in an environmental growth chamber (Thermoline CLIMATRON-520-SL-H, Australia) for a 72-h acclimatization period at 15 °C, under 12:12 h light: dark cycles (400 µmols/m^2^/second, measured 300 mm from light source). *Daphnia* were fed daily with 5 mL baker’s yeast suspension (1 g/L deionised water) and aquaria water was replaced with fresh spring water (~ 90%) (Tongariro Natural Spring Water, National Park, New Zealand; pH = 7.3, bicarbonate hardness ~ 117 mg/L) every other day. For our study, we collected a total of 96 macroinvertebrate predator individuals, comprising 48 backswimmers and 48 damselfly larvae. Backswimmers and damselfly larvae were collected from the same pond (36°57’37.8"S 174°55’53.0"E). Backswimmer larvae (8–13 mm in length) were collected from the water’s surface using a net (0.5 mm mesh); damselfly larvae (15–20 mm) were collected by sweeping a net through weedy vegetation near the pond’s edge. Subsequently, predators were placed in two 5 L aquaria containing spring water for 72-h under identical environmental conditions as *Daphnia*. To minimize natural variation in physiology, organisms were selected within narrow size ranges and within the same life history stage (larvae). Additionally, hunger levels were standardized by feeding predators *ad libitum* with live *Daphnia* for 48-h and then starving them for 24-h.

### Exposure 1: direct uptake of NP from the water column in prey and predators

We measured direct uptake of NP by prey and predators exclusively from the water column over a 24-h period. Prey and predators were not fed to prevent uptake of NP by feeding and duration was limited to 24-h to minimize physiological stress due to starvation. *Daphnia* and predators were exposed to NP in 250 mL glass beakers, each filled with 150 mL of spring water. We prepared 15 beakers for each organism, with each beaker holding 20 *Daphnia* or a single predator. Prior to introducing NP, we determined the baseline levels of Pd in organisms by harvesting three beakers for each species (3 × 20 *Daphnia*, 3 x each predator), which were subsequently prepared for ICP-MS analysis. We then introduced NP into the beakers at a concentration of 9 mg NP/L. The NPs were dispersed by pipetting the concentrated stock solution of NPs directly into the beaker, beneath the water surface to minimize surface tension effects, which could lead to particles accumulating at the air/water interface. The suspension was then gently stirred to ensure a homogeneous distribution of NPs throughout the entire water column. At 1, 6, 12, and 24 h post-addition, we collected and processed three individuals of each predator species. The organisms were rinsed with ultrapure water to remove adhering NP and prepared for ICP-MS analysis.

### Exposure 2: direct uptake of NP from the water column and indirect uptake from prey in predators

In Exposure 2, we measured the total uptake of NP by predators *directly* from water and *indirectly* by consumption of contaminated prey over a 5-day period. Subsequently, we measured the depuration of NP in predators over a 5-day period by feeding predators uncontaminated prey. Using these data, along with the uptake rate constants from water (*k*_*w*_) calculated in Exposure 1, uptake and depuration curves were generated for each predator using kinetic models.

Test beakers containing 150 mL spring water and 9 mg NP/L (see explanation for selected concentration at lines 313–319) were established and 20 *Daphnia* were added to each and left for 24-h (as outlined in Exposure 1) to provide time for them reach NP saturation. Subsequently, individual predators (previously unexposed to NP) were introduced to each beaker, accompanied by a 7 cm glass rod serving as a perch. Thirty-three beakers were prepared for each predator species. Each day during the 5-day uptake phase, predators were moved to fresh beakers containing NP, prey, and a glass rod to ensure a consistent level of prey exposure. Every 24 h, three individuals of each predator were randomly collected and subsequently prepared for ICP-MS analysis. This process was repeated for five days to evaluate the uptake of NP over time. On day 5, the remaining predators were transferred to beakers containing spring water with 20 unexposed *Daphnia* for a 5-day depuration phase. Three individuals of each predator species were harvested daily between days 6–10 and prepared for ICP-MS analysis to determine the depuration rate of NP.

### Sample digestion and NP quantification by ICP-MS

After collection, organisms were dried at 60 °C for 48 h, weighed and prepared for digestion. Samples were individually placed into 80 mL Teflon tubes and 4 mL HNO_3_ (69%; Surpapur, Merck), 1 mL HCl (37%; Suprapur, Merck), and 1 mL H_2_O_2_ (50%; Sigma Aldrich) were added. For every 20 samples, procedural blanks (4 mL HNO_3_, 1 mL HCl, 1 mL H_2_O_2_) were analysed for background Pd levels. Teflon tubes were sealed, placed in a Maxi-44 rotor, and digested in an Ethos-Up Microwave reaction system (Milestone SRL, Italy) at 200 °C for 20 min. The resulting digest was then cooled to room temperature, diluted with 45 mL ultrapure water, and a final weight obtained. ^105^Pd concentrations in the final solutions were quantitatively analysed on an Agilent 7700 ICP-MS in He mode to reduce polyatomic interferences. In our study, the isotopes ^105^Pd, ^106^Pd, and ^108^Pd had similar isotopic abundances, and thus any of these isotopes could be used to quantify the NPs. In this instance, we chose to use ^105^Pd to quantify NPs. Calibration standards were prepared in a matrix matched solution from 1000 mg/L single element standard (Inorganic Ventures, USA). A 20 µg/L Tb solution was added as an internal standard to monitor drift and matrix effects. Spike recovery tests were conducted on the invertebrates by adding a known concentration of NPs into the matrix to assess the effectiveness of the digestion protocol in recovering Pd. The recovery rate for triplicate samples of damselfly and backswimmer was 96.5 ± 0.5%, indicating the robustness and reproducibility of the extraction and analysis method. After obtaining Pd concentrations, we then back-calculated NP concentrations for each sample using the known metal: plastic ratio. The instrument limit of detection and limit of quantification (calculated as 10× the limit of detection) for Pd was 0.31 ng/L and 3.1 ng/L, respectively.

### Kinetic models to quantify uptake and depuration rates of NP

The concentration of NP in predators over time is a function of direct uptake from water, indirect uptake from prey, and depuration by predators. Despite our chosen NP concentration in both exposure assays (9 mg/L) exceeding natural environmental levels (up to 0.488 mg/L) [50], our study focuses on measuring NP transfer, not assessing ecotoxicological effects. Our transfer rate parameters are independent of the concentration in the water, prey and predators and can be applied to estimates of concentration in any given situation. Specific concentrations of NP are therefore not required; rather, an amount sufficient for tracking and measuring concentration in each compartment was essential. We determined direct uptake rate constants from Exposure 1 trials and depuration rate constants from the Exposure 2 trials. We then used these rate constants to solve for the rate of indirect NP uptake by predators from prey during the 5-day exposure phase. The depuration rate of NP was estimated using linear regression of log-transformed NP concentrations during the 5-day depuration phase (Eq. 1).

1 $$\:\frac{{C}_{predator}}{t}=\:-{k}_{d}{C}_{predator}$$

Where *C*_*predator*_ is the NP concentration in the predator (µg NP/mg DW predator), *t* is time and *k*_*d*_ is the depuration rate constant (d^− 1^).

NP concentrations in prey over time in Exposure 1 trials were fit with a Michaelis-Menten function, which has been used to model uptake of contaminants [51]. The maximum saturation concentration (*C*_*max*_) and the time taken to reach half the value of *C*_*max*_ (i.e., half saturation constant) were calculated using the “drc” package v3.0-1 in R [52].

The direct uptake rate of NP from water by predators was estimated using nonlinear least squares regression (nls) in base R, following Eq. 2 [13, 53]. Data were taken from the NP concentration in predators over time during Exposure 1.

2 $$\:{C}_{predator\:}=\frac{{k}_{w}{C}_{water}}{{k}_{d}}\:[1-{e}^{-{k}_{d}t}]\:$$

Where *k*_*w*_ is the uptake rate constant for NP from water (L/mg/d) and *C*_*water*_ is the concentration of NP in the water (µg/L). The value for *C*_*water*_ was assumed to remain constant over time and this is why the predators were moved to new beakers every 24-h.

We used *k*_*w*_ and *k*_*d*_ to determine indirect uptake rates from prey to predators using nonlinear least squares regression (nls) in base R, following Eq. 3 [53]. Data were taken from the NP concentration in predators over time during Exposure 2.

3 $$\:{C}_{predator\:}=\frac{{k}_{p}{C}_{prey}+{k}_{w}{C}_{water}}{{k}_{d}}\:[1-{e}^{-{k}_{d}t}]\:$$

Where *k*_*p*_ is the uptake rate constant for NP from the prey (mg prey/mg predator/d) and *C*_*prey*_ is the concentration of NP in the prey (µg NP/mg DW prey).

We checked the normality of model regression residuals using Shapiro-Wilk tests and used Q-Q plots to compare the distribution of the standardized residuals to a standard normal distribution. Confidence intervals for plots were estimated using the predFit() function in the “investr” package v1.4.2. All statistical analyses were conducted in R v4.2.1.

## Electronic supplementary material

Below is the link to the electronic supplementary material.

Supplementary Material 1

Supplementary Material 2

## Data Availability

No datasets were generated or analysed during the current study.
