# Supplementary material for: Predator traits influence uptake and trophic transfer of nanoplastics in aquatic systems–a mechanistic study
Source: Microplast nanoplast. 2024 Oct 9;4(1):20. doi: 10.1186/s43591-024-00096-4 (PMC11481666; doi:10.1186/s43591-024-00096-4)
Supplement: Supplementary file 1 — Supplementary Material 1 [file 43591_2024_96_MOESM1_ESM.docx]

**Supporting Information**

**Predator traits influence trophic transfer of nanoplastics in aquatic systems**

Αmy Ockenden^1*^, Denise M. Mitrano^2^, Melanie Kah^1^, Louis A. Tremblay^3,4^, Kevin S. Simon^1^

^1^ School of Environment, University of Auckland, Science Centre, Building 302, 23 Symonds Street, Auckland CBD, Auckland 1010, New Zealand

^2^ ETH Zurich, Department of Environmental Systems Science, Universitatstrasse 16, 8092 Zurich Switzerland

^3^ School of Biological Sciences, University of Auckland, Building 110, 3A Symonds Street, Auckland CBD, Auckland 1010, New Zealand

^4^ Cawthron Institute, 98 Halifax Street, The Wood, Nelson 7010, New Zealand

*Corresponding author:

Amy Ockenden: [a.ockenden@sheffield.ac.uk](mailto:a.ockenden@sheffield.ac.uk)


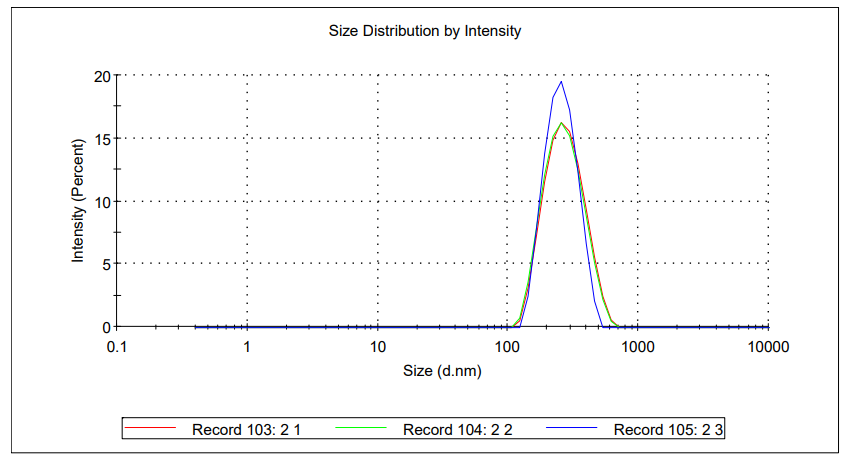


**Figure S1:** Size distribution report by intensity obtained from a Malvern Zetasizer. Mean hydrodynamic diameter of particles was confirmed to be 256.4 nm (PDI=0.113).

**Supplementary text 1: Calculating plastic:Pd ratio**

The plastic:Pd ratio was calculated by measuring the palladium concentration (mg/L) in the stock suspension using ICP-MS and determining the mass of nanoplastics (NPs) by drying 2 mL of the stock suspension at 60°C for 48 hours. The NP mass was measured in triplicate samples, yielding a value of 25,975 mg/L ± 2,338 mg/L (SD). The palladium concentration was also measured in triplicate samples using ICP-MS, resulting in 73.1 mg/L ± 0.14 mg/L. Consequently, the Pd to plastic ratio was 0.0028, corresponding to a palladium mass fraction of 0.28% (w/w).
